# Interplay of Pathogen-Induced Defense Responses and Symbiotic Establishment in *Medicago truncatula*

**DOI:** 10.3389/fmicb.2017.00973

**Published:** 2017-05-30

**Authors:** Tao Chen, Liujian Duan, Bo Zhou, Haixiang Yu, Hui Zhu, Yangrong Cao, Zhongming Zhang

**Affiliations:** ^1^State Key Laboratory of Agricultural Microbiology, Huazhong Agricultural UniversityWuhan, China; ^2^The Provincial Key Laboratory of Plant Pathology of Hubei Province, College of Plant Science and Technology, Huazhong Agricultural UniversityWuhan, China

**Keywords:** *Medicago truncatula*, *Sinorhizobium meliloti* Sm2011, *Pseudomonas syringae* pv. *tomato* DC3000, defense response, innate immunity, symbiosis

## Abstract

Suppression of host innate immunity appears to be required for the establishment of symbiosis between rhizobia and host plants. In this study, we established a system that included a host plant, a bacterial pathogen and a symbiotic rhizobium to study the role of innate immunity during symbiotic interactions. A pathogenic bacterium, *Pseudomonas syringae* pv. *tomato* strain DC3000 (*Pst* DC3000), was shown to cause chlorosis in *Medicago truncatula* A17. *Sinorhizobium meliloti* strain Sm2011 (Sm2011) and *Pst* DC3000 strain alone induced similar defense responses in *M. truncatula.* However, when co-inoculated, Sm2011 specifically suppressed the defense responses induced by *Pst* DC3000, such as MAPK activation and ROS production. Inoculation with Sm2011 suppressed the transcription of defense-related genes triggered by *Pst* DC3000 infection, including the receptor of bacterial flagellin (*FLS2*), pathogenesis-related protein 10 (*PR10*), and the transcription factor *WRKY33*. Interestingly, inoculation with *Pst* DC3000 specifically inhibited the expression of the symbiosis marker genes *nodule inception* and *nodulation pectate lyase* and reduced the numbers of infection threads and nodules on *M. truncatula* A17 roots, indicating that *Pst* DC3000 inhibits the establishment of symbiosis in *M. truncatula.* In addition, defense-related genes, such as *MAPK3/6*, *RbohC, and WRKY33*, exhibited a transient increase in their expression in the early stage of symbiosis with Sm2011, but the expression dropped down to normal levels at later symbiotic stages. Our results suggest that plant innate immunity plays an antagonistic role in symbiosis by directly reducing the numbers of infection threads and nodules.

## Introduction

Several genera of plants can benefit from atmospheric nitrogen fixation through symbiosis with particular microorganisms. The most widespread terrestrial mutualistic symbiosis is that between plants and arbuscular mycorrhizal fungi. The uptake of water and mineral nutrients (particularly phosphate) from soil by the fungal partner promotes the growth and disease resistance of the host plant (Parniske, 2008; Bonfante and Genre, 2010). A more typical symbiosis is that between most species of legume plants and N_2_-fixing bacteria, such as *Aminobacter*, *Azorhizobium*, *Bradyrhizobium*, *Devosia*, *Mesorhizobium*, *Methylobacterium*, *Microvirga*, *Ochrobactrum*, *Phyllobacterium*, *Rhizobium*, *Sinorhizobium*, *Burkholderia*, *Cupriavidus*, and *Herbaspirillum* (collectively called rhizobia) (Laranjo et al., 2014). The legume-rhizobium symbiosis results in the formation of a completely new organ, the root nodule, where the rhizobia are intracellularly hosted and fix atmospheric nitrogen into ammonia that can be assimilated by the plant (Oldroyd and Downie, 2008). There is a high specificity between a host legume species and its partner rhizobium in the establishment of a nitrogen-fixing symbiosis. For instance, the bacterium *Sinorhizobium meliloti* induces the formation of root nodules on *Medicago, Melilotus*, and *Trigonella*. *Mesorhizobium loti* is compatible with the *Lotus* species, whereas *Rhizobium etli* is compatible with species of the same genus (Gibson et al., 2008). NBS-LRR type disease resistance (R) genes are involved in determination of host specificity in the legume-rhizobia symbiosis (Yang et al., 2010). A successful interaction requires the strict coordination of two processes: rhizobium infection and nodule organogenesis (Oldroyd et al., 2011). Bacterial infection can occur either through root hair curls or cracks in the epidermis (Oldroyd and Downie, 2008). In most legumes, infection threads initiate from these infection sites, and allow the invasion of bacteria into the cortex. Finally, the cortical cells divide to form a nodule meristem, which further develops into a nodule (Kouchi et al., 2010). Symbiotic nitrogen fixation makes a major contribution to soil fertility and plays a critical role in sustainable agriculture. It is significant, as well as challenging, to transfer the establishment of such symbiosis to non-legumes for future agriculture development (Gewin, 2010). In rhizobial symbiosis, the establishment of a successful interaction requires the presence of signaling molecules called Nod factors (NFs) (Denarie and Cullimore, 1993). NFs are required for early steps of legume infection and root nodule organogenesis (Denarie et al., 1996). In addition, they are recognized by the LysM domain receptor kinases NFR1 and NFR5 in *Lotus japonicus* and LYK3 and NFP in *Medicago truncatula* (Amor et al., 2003; Limpens et al., 2003; Madsen et al., 2003; Radutoiu et al., 2003) to elicit calcium spiking and finally to reprogram the expression of specific symbiosis genes (Oldroyd, 2013). It has been reported that NFs also play a regulatory role in the production of reactive oxygen species (ROS) (Lohar et al., 2007; Cardenas et al., 2008). The mitogen-activated protein kinase (MAPK) cascade is associated with the NF signal transduction (Chen et al., 2012). *M. truncatula* NFP, initially described as a putative NF receptor, can also play a role in defense against pathogens, resistance to both the oomycete *Aphanomyces euteiches* and the fungus *Colletotrichum trifolii* (Rey et al., 2013).

Higher plants are continually exposed to various attacks, including bacteria, fungi, viruses, insects, and nematodes. To prevent invasion by these hostile microbes, plants appear to contain at least two branches of innate immune signaling: pathogen-associated molecular pattern-triggered immunity (PTI) and effector-triggered immunity (ETI) (Jones and Dangl, 2006). PTI is based on pattern recognition receptors (PRRs) that mediate recognition of microbe-associated molecular patterns (MAMPs). One of the best-characterized MAMP/PRR pair is flagellin/FLS2 in *Arabidopsis thaliana* (Zipfel et al., 2004; Boller and Felix, 2009). Flg22, a conserved 22-amino acid peptide from the bacterial flagellin protein, activates signaling cascade that includes calcium influx (Ranf et al., 2011), ROS production in surrounding infection sites (Torres et al., 2005), and activation of a MAPK cascade (Asai et al., 2002). Subsequently, defense responses, such as callose deposition, are induced (Clay et al., 2009). Flg22 signaling triggers the expression of many genes, such as WRKY transcription factors (Denoux et al., 2008). To avoid PTI, pathogens have evolved effector proteins that are directly delivered into the plant cell through translocation systems such as the bacterial types III and IV protein secretion systems (Feng and Zhou, 2012). R genes recognize these pathogen effector proteins according to the gene-for-gene theory (Block and Alfano, 2011). This form of resistance (termed as ETI) is often accompanied by a hypersensitive response at the infection site (Jones and Dangl, 2006). ETI or PTI receptors activate MAPK cascades that phosphorylate the downstream transcription regulators controlling the expression of early defense genes (van den Burg and Takken, 2010). Interestingly, some of the defense responses have also been detected in epidermal cells of legume roots in response to NF application (Ramu et al., 2002; Nakagawa et al., 2011), and the detected defense responses are transient and local (Cardenas et al., 2008; Jardinaud et al., 2016). *Bradyrhizobium japonicum* NFs have been found to suppress immune responses in both legumes and non-legumes (Liang et al., 2013). The overlap of defense and symbiotic signaling pathways was highlighted in a study of *L. japonicus* roots inoculated with its symbiotic partner *M. loti* and treated with the elicitor flg22 (Lopez-Gomez et al., 2012). There is increasing evidence that the perception of NFs might have evolved from chitin perception, which is related to PTI (Liang et al., 2014). Lipopolysaccharide (LPS) produced by some bacteria often acts as a virulence factor (Petrocelli et al., 2012). Purified LPS from *S. meliloti* can suppress an oxidative burst in *M. truncatula* a suspension of cells (Tellstrom et al., 2007). Almost all rhizobia can produce at least one type of exopolysaccharide (EPS) (Skorupska et al., 2006), and *S. meliloti* produces two types of EPS, namely succinoglycan and galactoglucan (Gibson et al., 2008). Transcriptomic data showed that a *S. meliloti* EPS-deficient mutant induced the expression of many defense-related genes in the *M. truncatula* host (Jones et al., 2008). Rhizobial surface polysaccharides (e.g., EPS, LPS, and cyclic glucans) have been implicated in facilitating infection thread formation and nodule development (Jones et al., 2008). It has been proposed that surface polysaccharides play a role in the evasion or suppression of host defense responses, a feature that is shared by pathogenic and symbiotic bacteria (D’Haeze and Holsters, 2004; Soto et al., 2009). Other strains, such as *Sinorhizobium* sp. NGR234, secrete a few translocate type III effectors including NopM, NopL, NopP, and NopT into host plant cells, where most of the effectors interfere with immune signaling pathways to suppress host defense responses (Skorpil et al., 2005; Dai et al., 2008; Xin et al., 2012; Ge et al., 2016).

*Pseudomonas syringae* pv. *tomato* DC3000 (*Pst* DC3000), a tomato pathogen of tomato (Cuppels, 1986), is also highly pathogenic to *A. thaliana* (Whalen et al., 1991). *Pst* DC3000 has been widely used to elucidate the general principles underlying plant immune response and bacterial pathogenesis (Xin and He, 2013; Yao et al., 2013). Plant roots are in contact with various microbes, including symbiotic and pathogenic microbes. However, how plants distinguish symbionts from pathogens is poorly understood. In this study, we successfully established a *Pst* DC3000-*M. truncatula* system, and the defense and symbiotic responses of *M. truncatula* plants were investigated. The interplay was studied by determining the expression of symbiosis marker genes and defense-related genes in plants co-inoculated with *S. meliloti* Sm2011 and *Pst* DC3000. These analyses were performed during the rhizobial infection and nodule formation on the roots and the initial hemibiotrophic colonization of *Pst* DC3000 on the leaves. It was found that the establishment of legume-rhizobium symbiosis suppresses the defense signaling pathways and that *Pst* DC3000 significantly reduces nodule organogenesis.

## Materials and Methods

### Plant Materials, Bacterial Strains, and Growth Conditions

*Medicago truncatula* genotype A17 was used in this study. Seedlings were grown as described in the Medicago Handbook^1^. In brief, seeds were scarified by immersion in concentrated H_2_SO_4_ for 3 min. After washing with sterile water, seeds were surface sterilized by immersion in 3% NaClO for 20 min, and germinated on 1.0% water agar plates at 20°C in the dark. Seeds were germinated under continuous light and cultured for 3 days. Ten-day-old seedlings grown on 1/2 Murashige and Skoog (MS) solid medium were used for a MAPK phosphorylation assay. The seedlings used for nodulation were planted in pots with sterile perlite and vermiculite at a 1:1 volume ratio supplemented with half-strength nitrogen-free Fahraeus medium (Fahraeus, 1957). Five-day-old seedlings were inoculated with approximately 10^7^ colony-forming units per mL wild type *S. meliloti* Sm2011 or GFP-labeled Sm2011 (carrying the pHC60 plasmid) (Chen et al., 2016). The numbers of infection threads and nodules were determined by using a fluorescence microscope.

### *Pst* DC3000 Infection of *M. truncatula*

*Pst* DC3000 was cultivated in the King’s B liquid medium supplemented with 75 μg mL^-1^ rifampicin at 28°C for overnight until the midlog growth phase (OD_600_ = 0.15) was reached (Lou et al., 2016). Bacteria were harvested by centrifugation at 2,500 × *g* for 10 min. The supernatant was discarded and bacteria were re-suspended in sterile water to a desired concentration. For spray inoculation, the bacterial suspension was adjusted to 5 × 10^8^ CFU/mL in water supplemented with 0.02% Silwet L-77. The surface of leaves was sprayed until they appeared to be evenly wet. Inoculated leaves were harvested at 3 days post inoculation (dpi) with forceps and fine-tipped scissors. Leaves were surface-sterilized by submerging in 70% ethanol for 10–15 s with gentle shaking. After washing with sterile water three times, each leaf was ground in a 1.5 mL tube with sterile distilled water using a cordless drill and a plastic micropestle until the tissue was completely macerated and no intact leaf pieces were visible. Serial dilutions were made and 100 μL of the samples were spotted on King’s B agar plates with appropriate antibiotics. Plates were incubated at 28°C for approximately 24–30 h or until the colonies were clearly visible. Colonies were identified by PCR and 16S rRNA sequencing. The identified colonies of *Pst* DC3000 were cultured as described above and prepared for spray inoculation of healthy *M. truncatula* leaves. The inoculated leaves (3 dpi) showed a diffuse chlorosis.

For inoculation of *M. truncatula* seedlings, bacterial suspension of *Pst* DC3000 (5 × 10^8^ CFU/mL supplemented with 0.02% Silwet L-77) was used for spray inoculation on leaves from 5-day-old seedlings in an ethanol-sterilized spray bottle set to release a fine mist. Spray the surface of the leaves until saturation (leaves appear evenly wet) (Yao et al., 2013). For co-inoculation of *M. truncatula* seedlings, the roots of 5-day-old seedlings were inoculated with *S. meliloti* Sm2011 first, followed by inoculation with *Pst* DC3000 as described above.

### MAPK Assay

MAPK assays were performed on roots or leaves of *M. truncatula* plants grown in 1/2 MS plates. The roots were cut into 0.5 cm pieces and the leaves were cut into 0.2 cm^2^ leaf disks, which were floated in petri dishes with phosphate buffer at pH 7.4 overnight at room temperature. For each treatment, 100 mg of roots or leaves were stimulated for 15 min with a cell suspension of bacteria (OD_600_ = 0.5). MAPK activation was monitored by western blot analysis. Proteins were separated using 12% PAGE and electro-blotted to a nitrocellulose membrane at 25 V for 40 min. The membrane was blocked with Tris-buffered saline plus Tween 20 containing 5% skim milk powder for 2 h at room temperature. After incubation with primary antibody and then with secondary antibody, the membrane was transferred for protein detection using a Thermo SuperSignal West Pico kit (Thermo Scientific). The sources and dilutions of antibodies were as follows: anti-phospho-p44/42 MAPK antibody (1: 2,500, Cell Signaling Technology, Beverly, MA, United States), actin protein antibody (1: 1000, Abmart, Shanghai, China), and goat anti-mouse horseradish peroxidase-conjugated antibody (1: 5000, Abmart, Shanghai, China).

### Detection of ROS

Reactive oxygen species production was detected using the fluorescent dye dichlorofluorescein (DCF) (Yao et al., 2015). To quantify pathogen-induced ROS levels, *M. truncatula* leaf disks (0.2 cm^2^) were excised from leaves and incubated in a 6-well-plate with water overnight, and then were treated with cell suspensions of *S. meliloti* Sm2011, *Pst* DC3000, *S. meliloti* Sm2011, and *Pst* DC3000 for 1 h. After treatment, leaf disks were loaded with 50 μM 2′, 7′-dichlorodihydrofluorescein diacetate (H_2_DCFDA) for 30 min (vacuum infiltrated for 15 min) and washed with ddH_2_O. All the pictures were taken using a fluorescence stereo microscope (Olympus SZX16, Tokyo, Japan) with excitation at 488 nm and emission at 525 nm to detect DCF fluorescence. The fluorescent images were analyzed with ImageJ software (National Institutes of Health, United States). Three independent biological replications were performed. For each treatment, 11–15 leaf disks were analyzed.

### Gene Expression Analysis

For studying the expression of *MAPK3* and *MAPK6* genes in a transient defense response (MAPK assay), RNA was isolated from *M. truncatula* root segments. For determining the expression of symbiosis- and defense-related genes in the plants, RNA was isolated from whole *M. truncatula* plants.

Total RNA was isolated using TRIZOL reagent (Invitrogen) and treated with DNase I (Promega), followed by extraction with phenol:chloroform (1:1). First-strand cDNA was synthesized from 500 ng of total RNA using an oligo(dT) primer. Quantitative real-time PCR reactions were performed using iTaq Universal SYBR Green SuperMix (BioRad) on a CFX96 real-time PCR system (BioRad) according to the manufacturer’s instructions. The thermal cycle was set as 95°C for 30 s, 95°C for 20 s, 60°C for 20 s, and 72°C for 20 s. The reaction was performed for 40 cycles. Three biological replicates were analyzed, and similar expression patterns were obtained. The *RBP1* (RNA-binding protein 1) gene was used as a constitutive control for the quantitative RT-PCR (Bourcy et al., 2013). Quantitative RT-PCR was used to examine the expression of *M. truncatula MtMAPK3* (gene ID: Medtr4g061130, Assembly: Mt4.0). *MtMAPK6* (gene ID: Medtr4g087620, Assembly: Mt4.0), *MtFLS2* (gene ID: Medtr4g094610.1, Assembly: Mt4.0), *MtNIN* (gene ID: Medtr5g099060.1, Assembly: Mt4.0), *MtNPL* (gene ID: Medtr3g086320.1, Assembly: Mt4.0), *MtRbohD* (gene ID: Medtr3g098320, Assembly: Mt4.0), *MtWRKY33* (gene ID: Medtr3g031220.1, Assembly: Mt4.0), *MtRbohC* (gene ID: Medtr3g098350, Assembly: Mt4.0), *MtPR10* (gene ID: Medtr2g035150.1, Assembly: Mt4.0), *MtPR5* (gene ID: 1g021945.1, Assembly: Mt4.0), *MtPR1* (gene ID: Medtr4g128750.1, Assembly: Mt4.0), *MtPAL* (gene ID: Medtr1g064090.1, Assembly: Mt4.0), *MtACTIN2* (gene ID: Medtr2g008050, Assembly: Mt4.0) and *oprf* gene of *P. syringae* (NC_004578.1). Primers are listed in Supplementary Table S1.

### Statistical Analysis

Each experiment was performed with more than three replicates and arranged in a complete random design. The data are presented as mean values ± SD (standard errors). Statistical analysis of experimental data was conducted using SAS (8.1) statistics software, was considered as significant when the *p*-value was <0.05 in a Student’s *t*-test.

## Results

### Establishment of Symbiosis and the Defense System

To study the plant response to different microbes, we set up an experimental system containing *M. truncatula* plants, the bacterial pathogen *Pst* DC3000, and the symbiont *S. meliloti* Sm2011. *M. truncatula* is one of the best model plants for studying symbiosis (Tang et al., 2014). The symbiotic interaction between *M. truncatula* and rhizobia resulted in the formation of nitrogen-fixing root nodules (**Supplementary Figures S1A,B**). In addition, *M. truncatula* could be infected by *Pst* DC3000 and shows disease symptoms, such as localized necrosis surrounded by diffuse chlorosis on the leaves (**Supplementary Figure S1C**). To confirm that the disease response is caused by *Pst* DC3000, the pathogenicity on *M. truncatula* was tested using Koch’s postulates (King, 1952). The clones isolated from the infected leaves were *Pst* DC3000, as indicated by PCR analysis, which were further used to inoculate healthy *M. truncatula* A17 leaves. As shown in **Supplementary Figure S1D**, leaves infected with *Pst* DC3000 or the isolated clones showed consistent disease phenotypes, which are similar to the phenotypes observed in *Arabidopsis* and tomato. These results indicate that *Pst* DC3000 can induce typical disease responses in *M. truncatula*.

### *S. meliloti* Sm2011 Suppressed MAPK Signaling Induced by *Pst* DC3000 in *M. truncatula*

**FIGURE 1:**
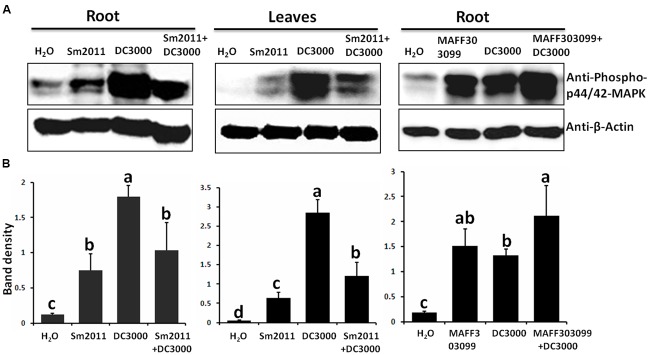
Inoculation with *Sinorhizobium meliloti* Sm2011 reduced *Pst* DC3000-induced mitogen-activated protein kinase (MAPK) phosphorylation in *Medicago truncatula* A17 roots and leaves. **(A)** *S. meliloti* Sm2011 specifically reduced *Pst* DC3000-induced MAPK phosphorylation. The roots or leaves of *M. truncatula* were treated with cell suspensions of *S. meliloti* Sm2011 (OD_600_ = 0.5), *Pst* DC3000 (OD_600_ = 0.5), *Mesorhizobium loti* MAFF303099 (OD_600_ = 0.5), a mixture of *S. meliloti* Sm2011 and *Pst* DC3000 (both concentration equivalent to OD_600_ = 0.5) or a mixture of *M. loti* MAFF303099 and *Pst* DC3000 (both concentration equivalent to OD_600_ = 0.5) for 15 min. Immunoblot analysis was performed using anti-phospho-p44/p42 MAPK antibody. The analysis of β-actin was performed as a loading control. **(B)** Intensity of the western blot signals **(A)** were quantitatively determined by ImageJ software (normalized MAP kinase levels of actin). The error bars represent SD values obtained from three biological replicates. Different letters indicate significant differences as determined by a *t*-tests (*p* ≤ 0.05).

To study the relationship between symbiosis and pathogenesis, cell suspensions of *Pst* DC3000 and/or *S. meliloti* Sm2011 were used to inoculate the leaves or roots of *M. truncatula* (**Figure 1A**). As activation of MAPK signaling culminates in expression of plant defense genes, phosphorylation of *M. truncatula* MAPKs was detected. The density of western blot bands was estimated by ImageJ software (**Figure 1B**). The results showed that the protein extracts from *M. truncatula* leaves or roots treated with *Pst* DC3000 and/or *S. meliloti* Sm2011 for 15 min activated MAPK phosphorylation (**Figure 1A**). However, the phosphorylation level of MAPKs induced by *S. meliloti* Sm2011 was lower than that induced by *Pst* DC3000 (**Figures 1A,B**). *M. truncatula* plants treated with both *S. meliloti* Sm2011 and *Pst* DC3000 showed a lower level of MAPK phosphorylation than those treated with *Pst* DC3000 alone (**Figures 1A,B**), suggesting that *S. meliloti* Sm2011 suppresses the *Pst* DC3000-triggered immunity in *M. truncatula.* To test whether the suppressive effect is specific to compatible symbionts, rhizobium strain *M. loti* MAFF303099, which is a symbiont for *L. japonicus* but not for *M. truncatula*, was used for inoculation. As shown in **Figures 1A,B**, *M. loti* MAFF303099 did not inhibit *Pst* DC3000-triggered MAPK activation. The effect of *S. meliloti* Sm2011 suppression was analyzed by comparing the kinetics of MAPK activation. We used leaves that were stimulated for 30 min and 1 h with a suspension of bacteria cells (**Supplementary Figure S2**). The results are consistent with leaves that were stimulated for 15 min (**Figure 1**). Expression analysis of *MAPK3* and *MAPK6* genes in roots 15 min post inoculation revealed slightly increased transcript levels (**Supplementary Figure S3**). These results suggest that *S. meliloti* Sm2011 specifically suppresses MAPK activation triggered by *Pst* DC3000.

### *S. meliloti* Sm2011 Reduced ROS Production Induced by *Pst* DC3000 in *M. truncatula*

**FIGURE 2:**
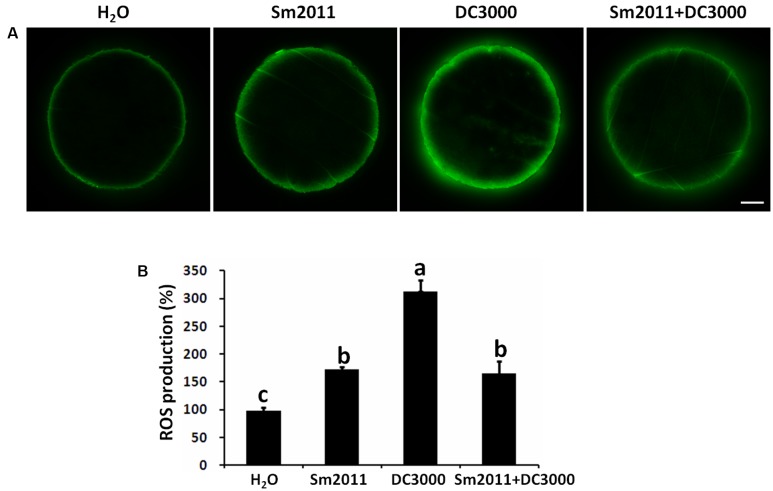
Inoculation with *S. meliloti* Sm2011 reduced *Pst* DC3000-induced reactive oxygen species (ROS) production in *M. truncatula* A17 leaves. **(A)** ROS production was detected with the fluorescent dye dichlorofluorescein (DCF). Leaf disks were treated with cell suspensions of *S. meliloti* Sm2011 (OD_600_ = 0.5), *Pst* DC3000 (OD_600_ = 0.5), or a mixture of *S. meliloti* Sm2011 and *Pst* DC3000 for 1 h, and then were incubated with 2′,7′-dichlorodihydrofluorescein diacetate (H_2_DCFDA) for 30 min. Scale bar = 1 mm. **(B)** Quantification of ROS levels in A. The error bars represent SD values obtained from three biological replicates. For each treatment, 11–15 leaf disks were analyzed. Different letters indicate significant differences as determined by a *t*-tests (*p* ≤ 0.05). The fluorescent intensity with H_2_O treatment was taken as 100%.

ROS levels were compared in leaf disks of *M. truncatula* treated with *S. meliloti* Sm2011 and/or *Pst* DC3000 cell suspensions. Significant ROS burst was observed after *Pst* DC3000 or Sm2011 treatment (**Figure 2**). The ROS level increased 1.8-fold after *S. meliloti* Sm2011 treatment. There was a 3.2-fold increase in ROS levels after *Pst* DC3000 treatment, whereas there was a 1.7-fold increase in ROS levels after *Pst S. meliloti* Sm2011 and *Pst* DC3000 treatment (**Figure 2**). This suggested that *S. meliloti* Sm2011 significantly reduced *Pst* DC3000-induced ROS production.

### Symbiosis- and Defense-Related Genes Were Affected by Co-inoculation of the Pathogen and Rhizobium

**FIGURE 3:**
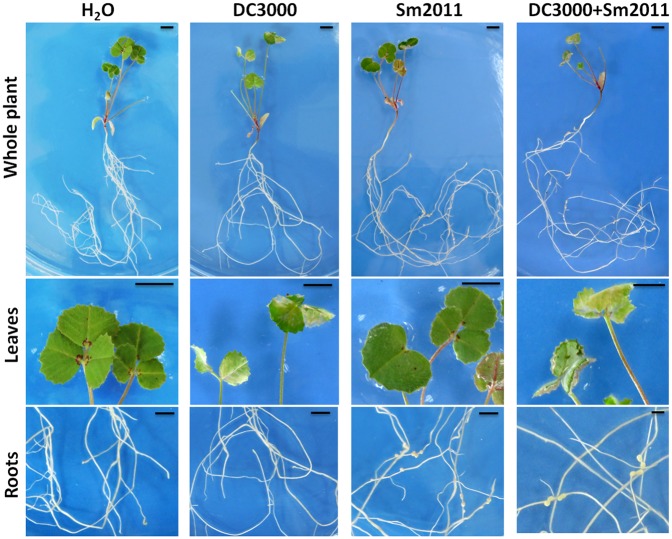
Images of *M. truncatula* infected by *S. meliloti* Sm2011 and *Pst* DC3000. *M. truncatula* plants that were inoculated with *S. meliloti* Sm2011, *Pst* DC3000, and co-inoculated with *S. meliloti* Sm2011 and *Pst* DC3000 at 8 days post inoculation (dpi). Bar = 0.5 cm.

**FIGURE 4:**
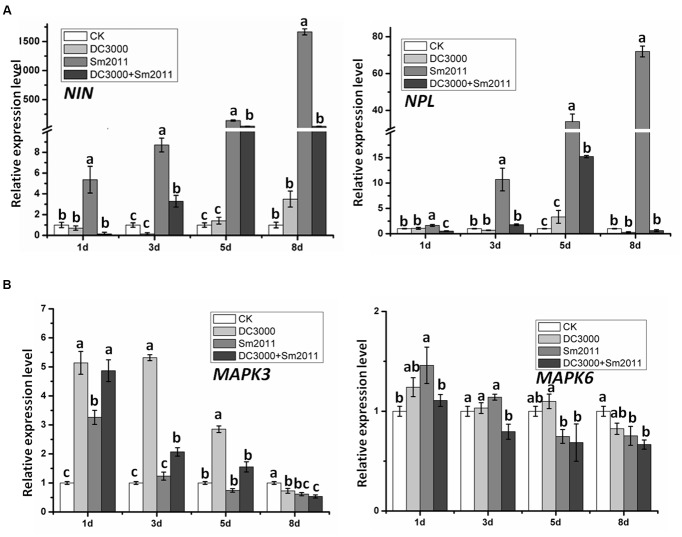
Quantitative RT-PCR analysis of symbiosis genes and MAPK gene expression patterns during early stages of nodule formation and *Pst* DC3000-induced disease. **(A)** Expression of *NIN* and *NPL* mRNA in total plants of *M. truncatula.* **(B)** Expression of *MAPK3* and *MAPK6* mRNA in *M. truncatula.* Total plants were harvested 1, 3, 5, and 8 dpi with *S. meliloti* Sm2011, *Pst* DC3000 or co-inoculation. Plants mock treated with water (uninoculated plants, CK) were also harvested at the same time intervals and served as a mock control. The RNA-binding protein (*RBP1*, GenBank: AJ508392.1) gene was used as an internal control (Bourcy et al., 2013). In all experiments, three independent replications were performed. The values are presented as the means ± SD. Different letters indicate significant differences as determined by a *t*-tests (*p* ≤ 0.05).

To further study the interplay between defense and symbiosis, expression patterns of symbiosis- and defense-related genes were investigated during early nodule formation and *Pst* DC3000 disease development. Nodule primordia and chlorosis symptoms became visible at 8 dpi (**Figure 3**). The symbiosis marker genes *nodule inception* (*NIN*) and *nodulation pectate lyase* (*NPL*), which are required for the development of infection threads and nodule primordia, were shown to be significantly induced by *S. meliloti* Sm2011 inoculation (**Figure 4A**). Co-application of *S. meliloti* Sm2011 and *Pst* DC3000 suppressed the transcript levels of *NIN* and *NPL* (**Figure 4A**). These findings suggest that the expression of symbiosis genes was suppressed by *Pst* DC3000-triggered immunity. The expression level of the *MAPK3* gene was increased at 1, 3, and 5 dpi with *Pst* DC3000, but dropped to the basal level at 8 dpi (**Figure 4B**). The expression of *MAPK6* slightly increased at 1 dpi with *S. meliloti*, and stayed at a low level at 3, 5, and 8 dpi (**Figure 4B**). *MAPK6* transcription was not induced by *Pst* DC3000 at 1, 3, and 5 dpi, and was even reduced at 8 dpi (**Figure 4B**). Levels of *MAPK6* increased at 1 dpi with *S. meliloti*, and were kept at a low level at 5 and 8 dpi (**Figure 4B**). These results suggest that *S. meliloti* Sm2011 caused a transient increase in the transcript levels of *MAPKs* during infection initiation and that there was a slight decrease in these transcripts when symbiosis was successfully established.

Plant NADPH oxidases, also known as respiratory burst oxidase homologs (RBOHs), have been identified as a major source of ROS during plant–microbe interactions (Daniel et al., 2012). Neither *Pst* DC3000 nor *S. meliloti* Sm2011 infection changed the expression pattern of *RbohD* in our experiments (**Supplementary Figure S4**). Instead, the expression of *RbohC* increased significantly at 3 dpi, but dropped to the basal level at 5 and 8 dpi in response to *Pst* DC3000 or *S. meliloti* Sm2011 infection (**Supplementary Figure S4**).

**FIGURE 5:**
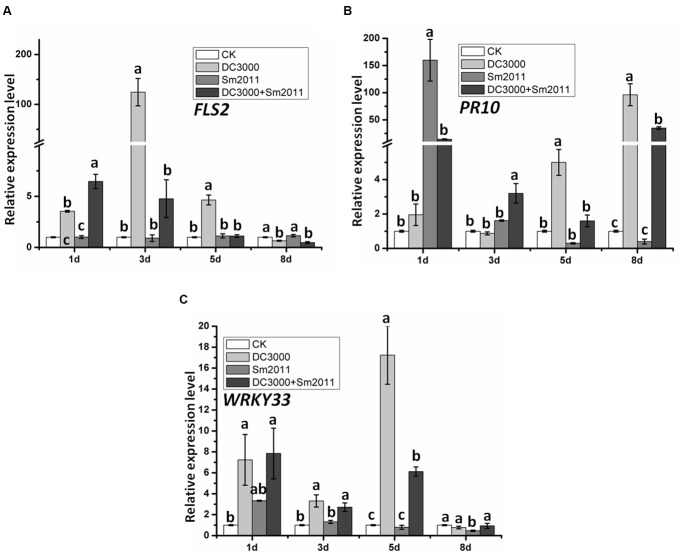
Quantitative RT-PCR analysis of defense-related gene expression patterns during the early stage of nodule formation and *Pst* DC3000 disease development. Expression of *FLS2* **(A)**, *PR10* **(B)**, and *WRKY33* **(C)**. mRNA in *M. truncatula*. Total plants were harvested 1, 3, 5 and 8 dpi with *S. meliloti* Sm2011, *Pst* DC3000 or co-inoculation. Plants treated with water (uninoculated plants) were also harvested at the same time intervals and served as the mock control. The *RBP1* gene was used as an internal control. In all experiments, three independent replications were performed. The values are presented as means ± SD. Different letters indicate significant differences as determined by a *t*-tests (*p* ≤ 0.05).

We further examined the expression patterns of other defense-related genes, such as *FLS2* (receptor of bacterial flagellin), pathogenesis-related protein 10 (*PR10*), and the *WRKY33* transcription factor. Expression of *FLS2* was significantly induced by *Pst* DC3000 infection but was unaltered by rhizobium infection (**Figure 5A**). *PR10* transcript level was significantly increased after 1 days of inoculation by *S. meliloti* Sm2011 (**Figure 5B**). The expression of *WRKY33* was significantly induced at first and then reduced at 8 dpi by *Pst* DC3000 infection, but it was transiently increased at 1 dpi with *S. meliloti* Sm2011 (**Figure 5C**). Co-inoculation of *S. meliloti* Sm2011 and *Pst* DC3000 resulted in the suppression of *FLS2, PR10*, and *WRKY33* transcription compared to *S. meliloti* Sm2011 and *Pst* DC3000 alone (**Figure 5**). These results indicate that *FLS2*, *PR10*, and *WRKY33* are associated with *Pst* DC3000 infection and *PR10* may also be related to nodulation.

**FIGURE 6:**
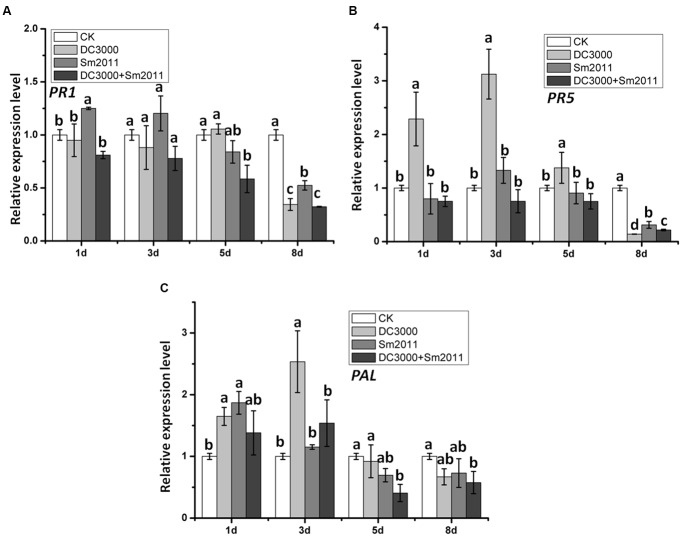
Relative expression of hormone signaling pathway marker genes *PR1*, *PR5*, and *PAL* during the early stage of nodule formation and *Pst* DC3000 disease development. Expression levels of *PR1* **(A)**, *PR5* **(B)**, and *PAL* **(C)** mRNA in *M. truncatula.* Total plants were harvested 1, 3, 5, and 8 dpi with *S. meliloti* Sm2011, *Pst* DC3000 or co-inoculation. Plant a mock treated with water (uninoculated plants, CK) was harvested at the same time and served as a control. In all experiments, three independent replications were performed. The values are presented as the means ± SD. Different letters indicate significant differences as determined by a *t*-tests (*p* ≤ 0.05).

We detected the expression levels of the SA-signaling marker gene *PR1* and *PR5* under treatment conditions. No significant change in the transcripts level of *PR1* was observed between *Pst* DC3000 and *S. meliloti* Sm2011 infection, while the expression of *PR5* increased significantly at 1, 3, and 5 dpi with *Pst* DC3000 strains and is lower in co-inoculated plants than in those inoculated with *Pst* DC3000 alone (**Figures 6A,B**). L-Phenylalanine ammonia-lyase (PAL) is the first enzyme involved in the phenylpropanoid biosynthesis pathway (Dixon et al., 2002). The expression of *PAL* increased significantly at 1 and 3 dpi and reduced to base level with *Pst* DC3000 strains (**Figure 6C**).

### *Pst* DC3000 Inhibited Establishment of Symbiosis between *M. truncatula* and *S. meliloti*

**FIGURE 7:**
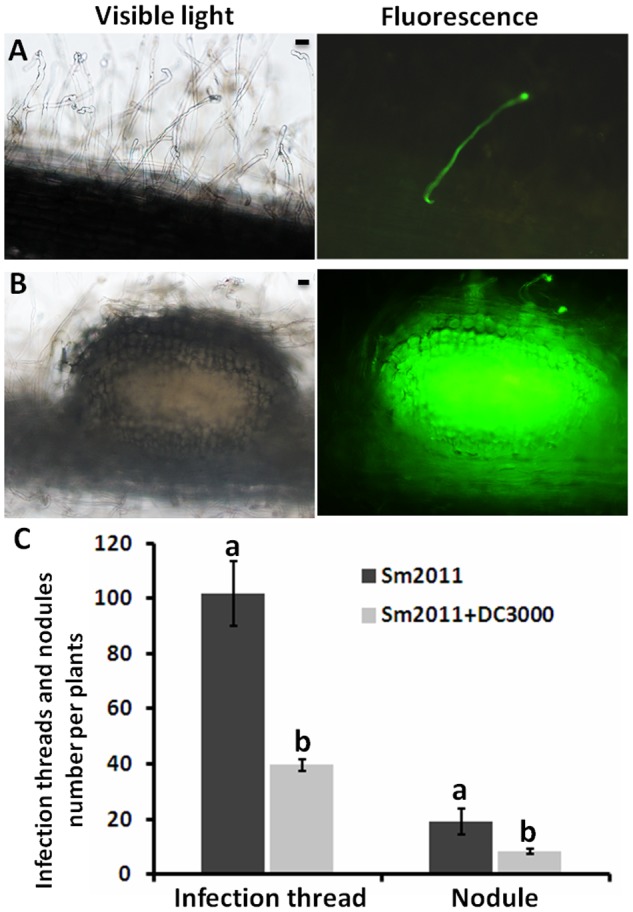
Numbers of infection threads and nodule primordia per plant in *M. truncatula* inoculated with GFP-labeled *S. meliloti* Sm2011 and/or *Pst* DC3000. **(A)** Infection thread formation in the roots of *M. truncatula* inoculated with GFP-labeled *S. meliloti* Sm2011 and *Pst* DC3000 at 5 dpi, bar = 20 μm. **(B)** Nodule primordia formation in the roots of *M. truncatula* inoculated with GFP-labeled *S. meliloti* Sm2011 and *Pst* DC3000 at 8 dpi, bar = 20 μm. **(C)** Numbers of infection threads (at 5 dpi) and nodules (at 8 dpi) per root of the wild type R108 inoculated with GFP-labeled *S. meliloti* Sm2011 and/or *Pst* DC3000. The values are presented as the means ± SD. Different letters indicate significant differences as determined by *t*-tests (*p* ≤ 0.05).

The effect of *Pst* DC3000 on the establishment of symbiosis between *M. truncatula* and *S. meliloti* was analyzed during different stages of nodule formation. The amounts of infection threads and nodule primordia per plant were calculated after inoculation with GFP-labeled *S. meliloti*. Infection threads were visualized under a fluorescence microscope (**Figure 7A**), and a 61% reduction was observed upon co-inoculation with *Pst* DC3000 and *S. meliloti* Sm2011 at 5 dpi (**Figure 7C**). After 8 days, the nodule primordia and nodules became visible in plants (**Figure 7B**), and a 57% reduction in nodules was observed when *Pst* DC3000 was co-inoculated (**Figure 7C**). These results suggest that *Pst* DC3000 significantly inhibits nodule organogenesis in *M. truncatula.*

### *S. meliloti* Sm2011 Inhibited *Pst* DC3000 Growth

**FIGURE 8:**
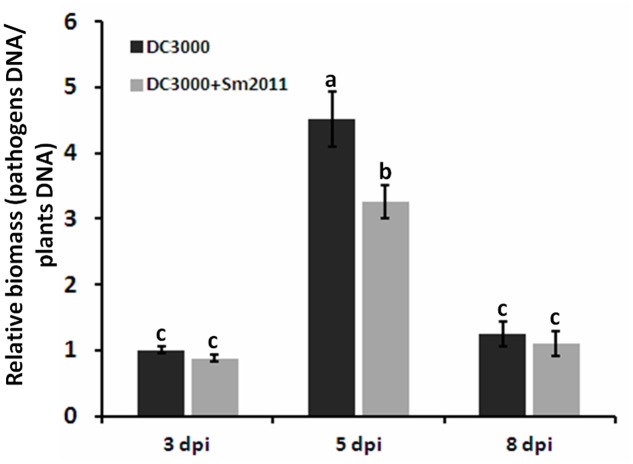
The growth of *Pst* DC3000 was determined by qPCR-based biomass. Disease progression of on *M. truncatula* plants inoculated with *Pst* DC3000 and/or *S. meliloti* Sm2011 at 3, 5, and 8 dpi. For qPCR analysis about 30 ng of DNA were mixed with 0.4 mM gene specific primers [bacterial biomass: *oprf* gene (Ross and Somssich, 2016), plant biomass: *actin*]. Normalized plant DNA of *actin* and normalized pathogen DNA of *oprf*, pathogen or plant DNA is related to plant biomass. The error bars indicate standard deviations of three independent biological replicates. Different letters indicate significant differences as determined by a *t*-tests (*p* ≤ 0.05).

The effect of *S. meliloti* Sm2011 on the pathogen invasion between *M. truncatula* and *Pst* DC3000 was analyzed with qRT-PCR analysis to determination of bacterial growth. The quantiication of *Pst* DC3000 by highly sensitive DNA-based methods like qRT-PCR has been reported (Brouwer et al., 2003; Ross and Somssich, 2016). Significantly reduction in bacterial growth of *Pst* DC3000 was observed when *Pst* DC3000 and *S. meliloti* Sm2011 were co-inoculated at 5 dpi (**Figure 8**). The results suggested that *S. meliloti* Sm2011 significantly inhibits bacterial growth in the leaves of *M. truncatula.*

## Discussion

Legumes can establish an efficient symbiotic interaction with rhizobia, which results in the production of nodules. However, defense responses are inevitably activated when plants are attacked by pathogens. Thus, a mechanism to successfully avoid or resist plant immune responses is a prerequisite for the establishment of symbiosis between the host and symbiotic rhizobia. In this study, we established a convenient system, to study the interplay between immunity and symbiosis, which consists of *M. truncatula* A17 plants, pathogenic bacterium *Pst* DC3000 and rhizobia.

The phosphorylation levels of MAPK3 and MAPK6 were elevated by *Pst* DC3000 as well as by *S. meliloti* Sm2011 (**Figure 1A**), suggesting that the phosphorylation of MAPK3 and MAPK6 is associated with defense and symbiotic responses. However, the phosphorylation level of MAPK3 and MAPK6 induced by rhizobia was lower than that induced by *Pst* DC3000. Importantly, inoculation with compatible rhizobia could suppress the pathogen-triggered MAPK phosphorylation as shown by three time-course experiments (**Figure 1** and **Supplementary Figure S2**). In addition, treatment of *M. truncatula* leaves with *S. meliloti* Sm2011 and *Pst* DC3000 significantly reduced *Pst* DC3000-induced ROS production (**Figure 2**). These results suggest that rhizobia produce elicitors (unknown MAMPs) that are detected by the host plant resulting in suppressed *Pst* DC3000-triggered immunity in *M. truncatula.* Bacterial cell surface components, such as oligo- and polysaccharides, are considered as crucial signals for the interactions of bacteria with host plants (Mithofer, 2002). In addition to effectors, several signals from rhizobia have been identified to be associated with the suppression of the host defense in legume roots, including NFs, LPS, and EPS (Luo and Lu, 2014; Gourion et al., 2015; Tóth and Stacey, 2015; Yasuda et al., 2016). It has been proposed that the symbiotic suppression of legume innate immunity is a result of co-evolution (Gourion et al., 2015) and that the identified signals for symbiosis may interact with immune signaling pathways to suppress host defense responses.

To gain a better understanding of the possible overlap of molecular mechanisms underlying symbiosis and disease development, we investigated the expression patterns of symbiosis- and defense-related genes during the early symbiotic stage and disease development caused by *Pst* DC3000 (**Figures 4**, **5** and **Supplementary Figure S4**). The expression of *NIN* and *NPL* was lower in co-inoculated roots than in those inoculated with *S. meliloti* Sm2011 alone (**Figure 4A**). These results suggest that the symbiotic pathway is suppressed when defense signaling pathways are activated. Similarly, the expression of *FLS2*, *PR10*, and *WRKY33* was lower in co-inoculated plants than in those inoculated with *Pst* DC3000 alone (**Figure 5**), suggesting that defense signaling pathways are suppressed during the establishment of symbiosis. In addition, the transcript levels of defense-related genes *MAPKs*, *RbohC*, and *WRKY33* exhibited a transient increase in response to rhizobial inoculation, whereas low levels were measured during the formation of nodule primordia (**Figures 4B**, **5C** and **Supplementary Figure S4**). Hence, a rapid and defense-like response occurred in *M. truncatula* upon *S. meliloti* inoculation. These findings are consistent with previous speculations (Tóth and Stacey, 2015).

Plant immune responses are usually accompanied by the production of ROS, one of the earliest responses following pathogen infection in plants. Genetic and biochemical evidence indicates that NADPH oxidases (RBOHs) are key compounds for ROS production involved in plant innate immunity (Arthikala et al., 2014). RBOHs have been described as a major source of ROS during the establishment of root nodules (Puppo et al., 2013). In this study, no significant change in the transcripts level of *RbohD* was observed between *Pst* DC3000 and *S. meliloti* Sm2011 infection, while the expression of *RbohC* increased significantly at 3 dpi with both strains (**Supplementary Figure S3**). It is possible that another RBOH gene should be used for detection.

Defense and symbiotic responses in *M. truncatula* appear to influence each other. *Pst* DC3000 likely caused a negative effect on the symbiotic interaction between *M. truncatula* and *S. meliloti.* This negative effect resulted in a decrease in infection threads and reduced nodulation (**Figure 7**). *Pst* DC3000 affected nodule organogenesis. *Pst* DC3000 caused diffuse chlorosis on leaves, and thus, likely had reduced photosynthesis and uptake of nutrients. In this view, poor nodulation would be the result of weak plant growth. Legumes possess a systemic negative feedback regulatory system called autoregulation of nodulation (AON), which controls the nodule number and the nodulation zone through long-distance root-shoot signaling (Oka-Kira and Kawaguchi, 2006; Ferguson et al., 2010). AON is initiated during nodule development by the synthesis of a root-derived signal named ‘Q’ (Okamoto et al., 2009). *Pst* DC3000 infected plant leaves and *S. meliloti* Sm2011 infected plant roots. Maybe there is Q signal by the AON in the leaf results in the production of a novel shoot-derived inhibitor, which appears to enter the phloem and travels down to the root where it acts to inhibit further nodulation events (Lin et al., 2010). The involvement of hormone in AON (Kinkema and Gresshoff, 2008). Plant hormones are likely to be essential throughout nodule organogenesis for integration of developmental and environmental signaling cues into nodule development (Ryu et al., 2012; Ferguson and Mathesius, 2014). Under the condition of pathogen attack, plants generated salicylic acid (SA) and jasmonic acid (JA) acting as secondary signaling molecules to modulate plant defense response. Indeed, the expression of defense-related genes *PR5* and *PAL* were significantly induced when plants were inoculated with *Pst* DC3000, while co-inoculation with *Pst* DC3000 and *S. meliloti* Sm2011decreased the expression levels of *PR5* and *PAL* (**Figure 6**). These data confirmed that rhizobia repress immune response triggered by bacterial pathogens.

Tripartite legume/rhizobium/pathogen interactions are complex systems and their analysis will require future attention. In our test system, it is hard to monitor the number of mature nodules (2 or 3 weeks post inoculation) since *Pst* DC3000 could cause the death of *M. truncatula* leaves and seriously affected the growth of plants, which impaired nodule development. Instead, we took advantage of quantitative PCR to compare the growth of *Pst* DC3000 in plants in response to rhizobia treatment. The quantification of *Pst* DC3000 by DNA-based technique is highly reliable and comparable (Brouwer et al., 2003; Ross and Somssich, 2016). *S. meliloti* Sm2011 significantly inhibits bacterial growth in the leaves of *M. truncatula* (**Figure 8**). Besides, some of leaves died (because of the pathogen infection) and fell from the plants may result in lower bacterial growth at 8 dpi than at 5 dpi.

Rhizobia treatment inhibits both growth of *Pst* DC3000 and defense-related genes expression. The possible mechanism involved in this is that rhizobia may inhibit the growth of *Pst* DC3000 through an unknown mechanism or compete with *Pst* DC3000 to infect plants leading to lower number of *Pst* DC3000 grown in plants. The reduced number of *Pst* DC3000 might be the direct reason to induce less immune response in plants leading to decreased defense-related gene expression.

Various *M. truncatula* mutants have been characterized, especially symbiosis mutants. Future gene expression analysis of these mutants inoculated with *Pst* DC3000 will further enhance our understanding on regarding the overlap of molecular mechanisms underlying symbiosis and disease development.

## Author Contributions

TC and ZZ designed the research and wrote the paper. TC, BZ, and LD executed the experiments. TC, BZ, LD, HY, HZ, YC, and ZZ performed the data and analyses. All authors read and approved the final manuscript.

## Conflict of Interest Statement

The authors declare that the research was conducted in the absence of any commercial or financial relationships that could be construed as a potential conflict of interest.

## Footnotes

1 http://www.noble.org/medicagohandbook

## Supplementary Material

FIGURE S1 Nodulation and disease symptoms of *M. truncatula*. **(A)** Photograph of 40-day-old *M. truncatula* plants. Bar = 0.5 cm. **(B)** Nodulation phenotypes of *M. truncatula.* Bar = 0.5 cm. **(C)** Leaves showing disease symptoms at 5 dpi with *Pst* DC3000. Bar = 0.5 cm. **(D)** Disease symptoms of *M. truncatula* at 5 dpi with *Pst* DC3000. Bacteria were re-isolated from infected leaves. Bar = 0.5 cm.

FIGURE S2 Activation of MAPK phosphorylation in roots/leaves of *M. truncatula* in response to treatments with a cell suspension for 30 min or 60 min. **(A)** The treatments with a cell suspension including *S. meliloti* Sm2011 (OD_600_ = 0.5), *Pst* DC3000 (OD_600_ = 0.5), *M. loti* MAFF303099 (OD_600_ = 0.5), a mixture of *S. meliloti* Sm2011 and *Pst* DC3000 (both concentration equivalent to OD_600_ = 0.5) or a mixture of *M. loti* MAFF303099 and *Pst* DC3000 (both concentration equivalent to OD_600_ = 0.5). Immunoblot analysis was performed using anti-phospho-p44/p42 MAPK antibody. The analysis of β-actin was performed to show equal loading. **(B)** Intensity of the western blot signals **(A)** were quantitatively determined by ImageJ software (normalized MAP kinase levels of actin). The error bars represent SD values obtained from three biological replicates. Different letters indicate significant differences as determined by a *t*-tests (*p* ≤ 0.05).

FIGURE S3 Quantitative RT-PCR analysis of *MAPK3* and *MAPK6* transcripts in roots of *M. truncatula* treated with cell suspensions of *S. meliloti* Sm2011, *Pst* DC3000 or a mixture of both strains for 15 min. The housekeeping gene *actin* was used as an internal control. The error bars represent SD values obtained from three biological replicates. Different letters indicate significant differences as determined by a *t*-tests (*p* ≤ 0.05).

FIGURE S4 Quantitative RT-PCR analysis of NADPH oxidase genes during the early stage of nodule formation and *Pst* DC3000 disease development. Expression of *RbohD* and *RbohC* mRNA in *M. truncatula.* Total plants were harvested 1, 3, 5, and 8 dpi with *S. meliloti* Sm2011, *Pst* DC3000 or co-inoculation. Plant a mock treated with water (uninoculated plants, CK) was harvested at the same time and served as a control. In all experiments, three independent replications were performed. The values are presented as the means ± SD. Different letters indicate significant differences as determined by a *t*-tests (*p* ≤ 0.05).
