# Supplementary figures and images for: Interplay of Pathogen-Induced Defense Responses and Symbiotic Establishment in Medicago truncatula
Source: Front Microbiol. 2017 May 30;8:973. doi: 10.3389/fmicb.2017.00973 (PMC5447765; doi:10.3389/fmicb.2017.00973)

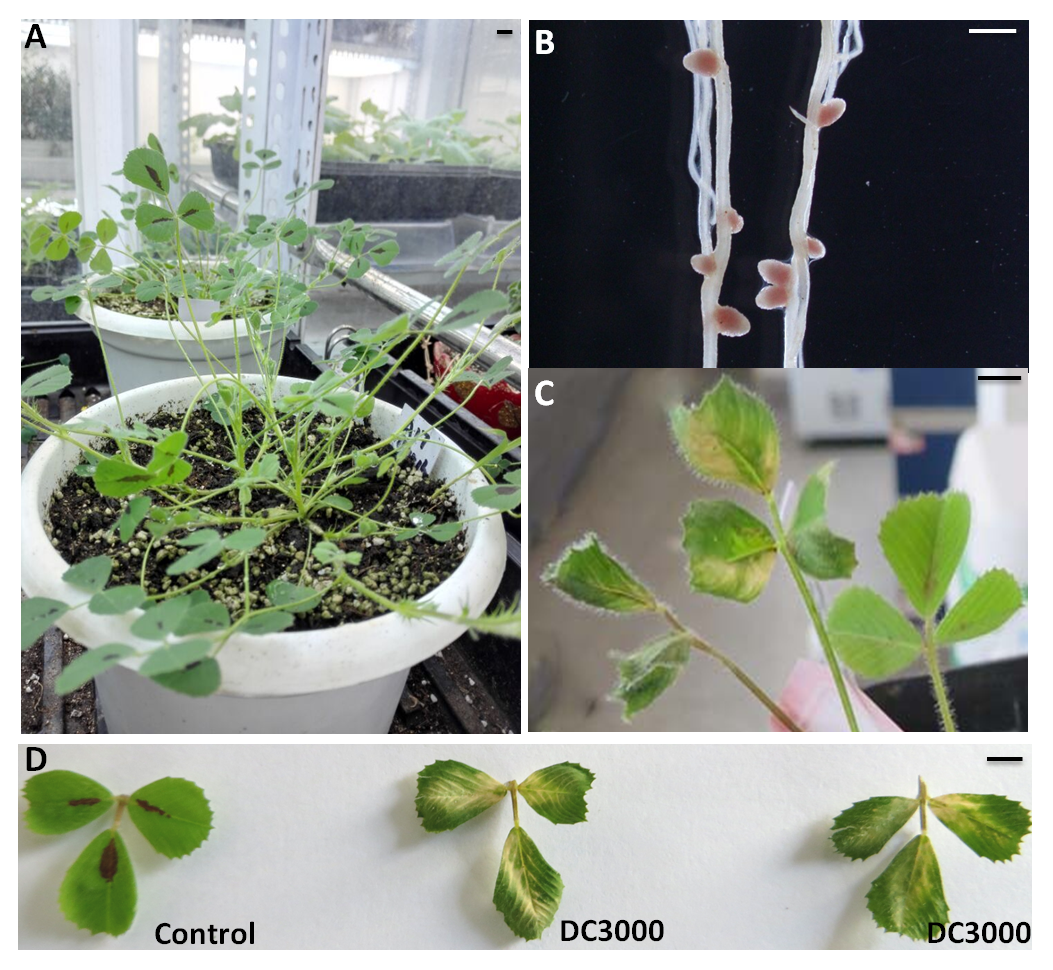

Supplement: FIGURE S1 — Nodulation and disease symptoms of M. truncatula. (A) Photograph of 40-day-old M. truncatula plants. Bar = 0.5 cm. (B) Nodulation phenotypes of M. truncatula. Bar = 0.5 cm. (C) Leaves showing disease symptoms at 5 dpi with Pst DC3000. Bar = 0.5 cm. (D) Disease symptoms of M. truncatula at 5 dpi with Pst DC3000. Bacteria were re-isolated from infected leaves. Bar = 0.5 cm. [file Image_1.TIF]

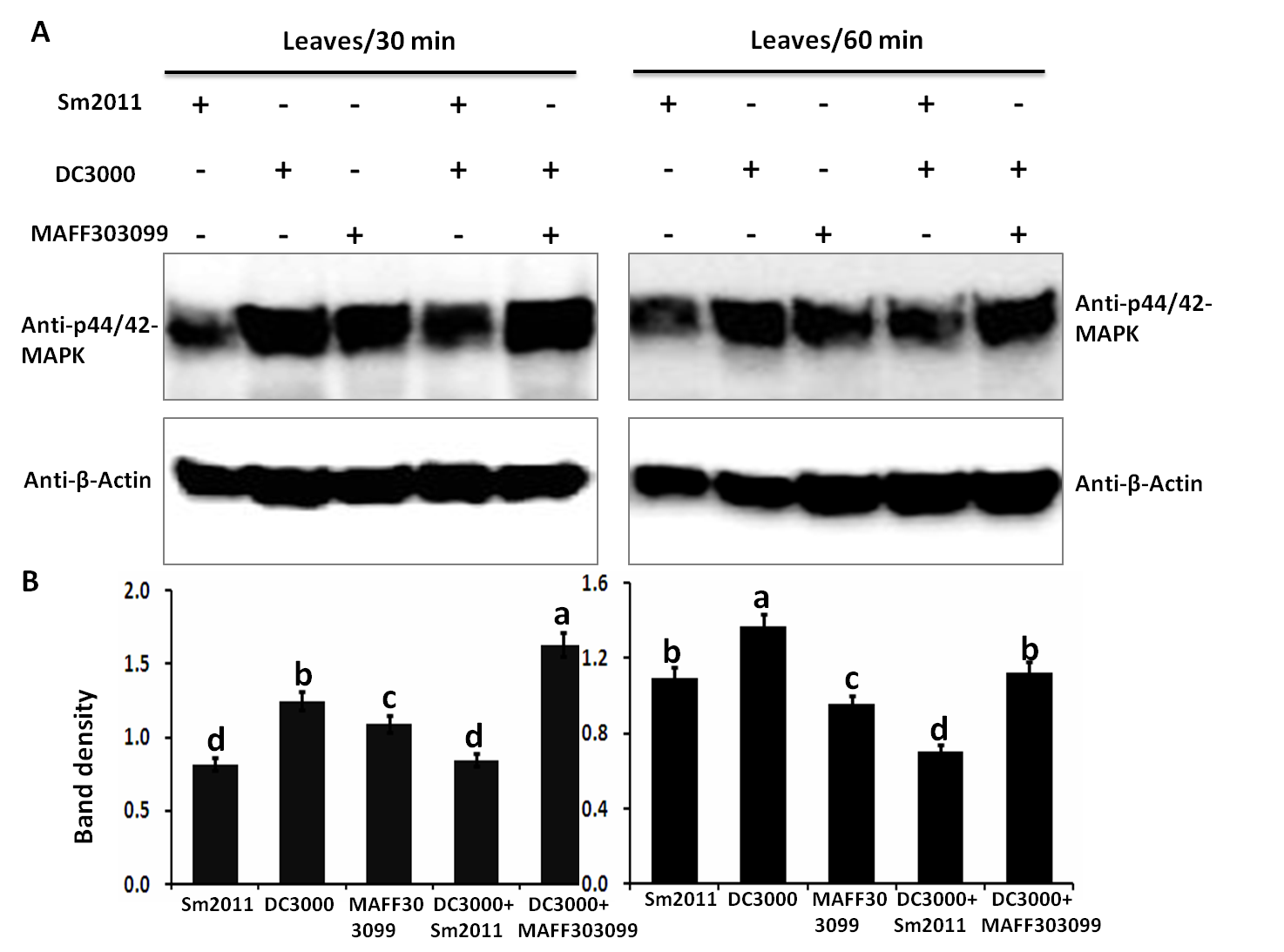

Supplement: FIGURE S2 — Activation of MAPK phosphorylation in roots/leaves of M. truncatula in response to treatments with a cell suspension for 30 min or 60 min. (A) The treatments with a cell suspension including S. meliloti Sm2011 (OD600 = 0.5), Pst DC3000 (OD600 = 0.5), M. loti MAFF303099 (OD600 = 0.5), a mixture of S. meliloti Sm2011 and Pst DC3000 (both concentration equivalent to OD600 = 0.5) or a mixture of M. loti MAFF303099 and Pst DC3000 (both concentration equivalent to OD600 = 0.5). Immunoblot analysis was performed using anti-phospho-p44/p42 MAPK antibody. The analysis of β-actin was performed to show equal loading. (B) Intensity of the western blot signals (A) were quantitatively determined by ImageJ software (normalized MAP kinase levels of actin). The error bars represent SD values obtained from three biological replicates. Different letters indicate significant differences as determined by a t-tests (p ≤ 0.05). [file Image_2.TIF]

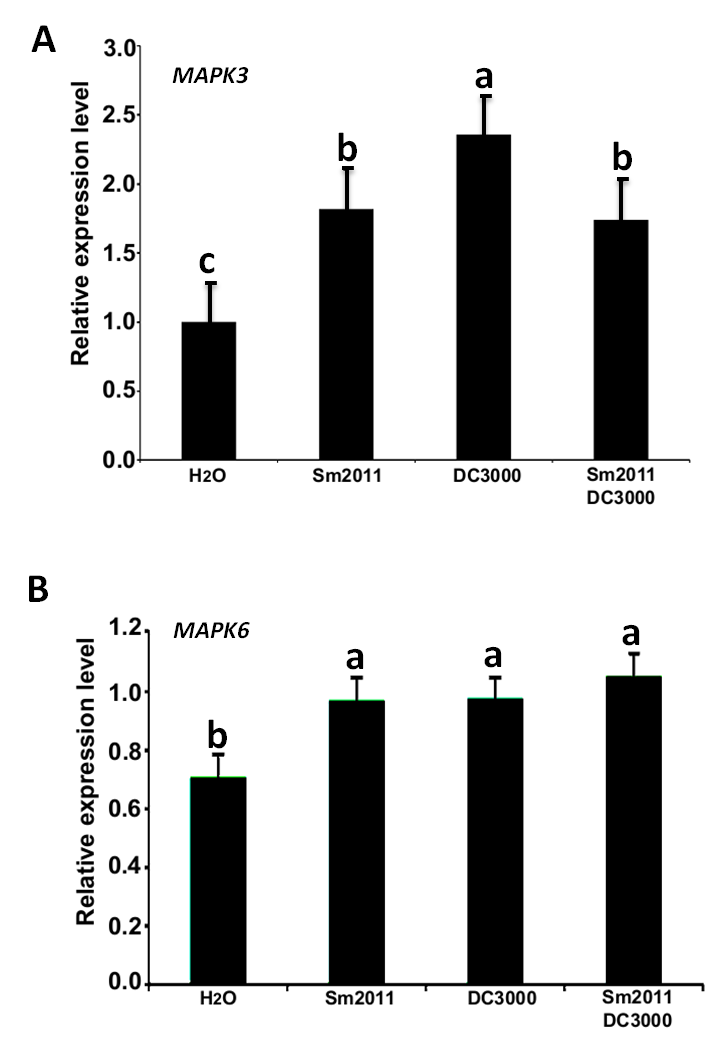

Supplement: FIGURE S3 — Quantitative RT-PCR analysis of MAPK3 and MAPK6 transcripts in roots of M. truncatula treated with cell suspensions of S. meliloti Sm2011, Pst DC3000 or a mixture of both strains for 15 min. The housekeeping gene actin was used as an internal control. The error bars represent SD values obtained from three biological replicates. Different letters indicate significant differences as determined by a t-tests (p ≤ 0.05). [file Image_3.TIF]

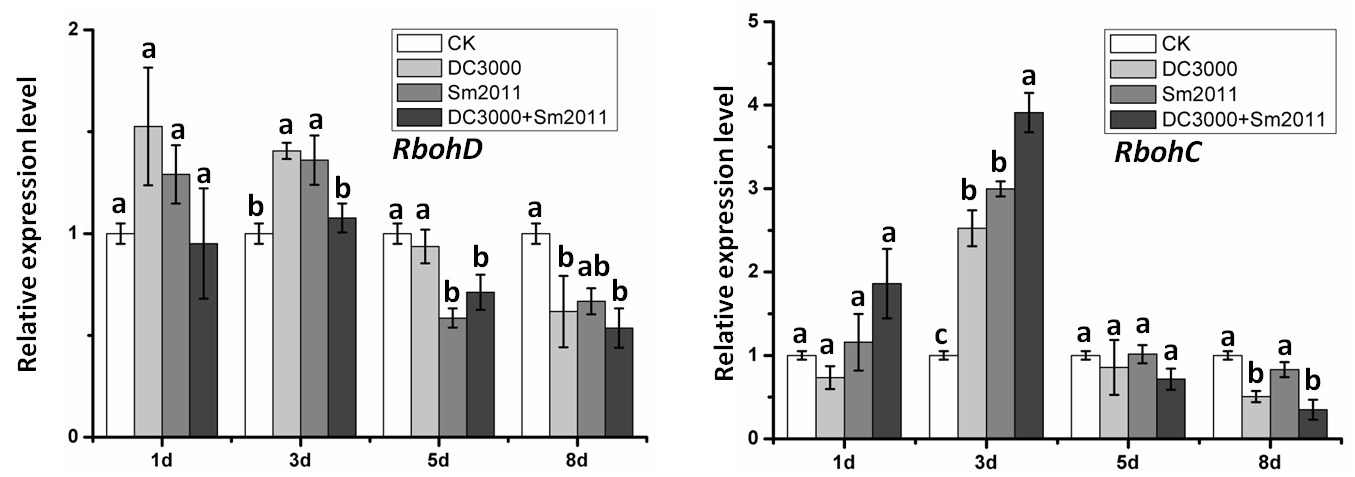

Supplement: FIGURE S4 — Quantitative RT-PCR analysis of NADPH oxidase genes during the early stage of nodule formation and Pst DC3000 disease development. Expression of RbohD and RbohC mRNA in M. truncatula. Total plants were harvested 1, 3, 5, and 8 dpi with S. meliloti Sm2011, Pst DC3000 or co-inoculation. Plant a mock treated with water (uninoculated plants, CK) was harvested at the same time and served as a control. In all experiments, three independent replications were performed. The values are presented as the means ± SD. Different letters indicate significant differences as determined by a t-tests (p ≤ 0.05). [file Image_4.TIF]
